# 5 Alpha Reductase Inhibitors (5ARIs) Monotherapy and Combinations: Current Role in Benign Prostatic Hyperplasia (BPH) Management

**DOI:** 10.3390/medicina62050975

**Published:** 2026-05-17

**Authors:** Christos Roidos, Petros Sountoulides, Konstantinos Papathanasiou, Asterios Symeonidis, Ioannis Mykoniatis

**Affiliations:** 1^st^ Department of Urology, School of Medicine, Faculty of Health Sciences, Aristotle University of Thessaloniki, 541 24 Thessaloniki, Greece; drchriroid22@gmail.com (C.R.); papathanasioukonstantinos@hotmail.com (K.P.); symeaste@gmail.com (A.S.); g_mikoniatis@hotmail.com (I.M.)

**Keywords:** benign prostatic hyperplasia, 5-a reductase inhibitors, α-blockers, BPH progression, dutasteride, finasteride, combination therapy

## Abstract

*Background and Objectives*: Benign prostatic hyperplasia (BPH) is a progressive, androgen-dependent condition driven by dihydrotestosterone (DHT). 5α-reductase inhibitors (5-ARIs), including finasteride and dutasteride, target this pathway and provide disease-modifying effects. *Materials and Methods*: This narrative review summarizes evidence from randomized trials, meta-analyses, and observational studies evaluating the efficacy, timing, and safety of 5-ARIs in the management of LUTS due to BPH. *Results*: 5-ARI therapy reduces prostate volume by 18–28% and serum PSA by approximately 50% within 6–12 months. Landmark trials (MTOPS, CombAT) demonstrate significant reductions in acute urinary retention (AUR) and BPH-related surgery (>50% RR reduction). Combination therapy with α-blockers provides superior symptom control and greater prevention of clinical progression, particularly in men with prostate volume ≥ 30–40 mL or PSA ≥ 1.5 ng/mL. Early initiation of combination therapy improves long-term outcomes, while α-blocker withdrawal may be feasible in selected patients. Adverse events are mainly sexual, with emerging data suggesting a possible association with depression. *Conclusions*: 5-ARIs are central to BPH management, offering sustained clinical benefits and prevention of progression. Optimal outcomes depend on appropriate patient selection, early treatment in high-risk individuals, and individualized long-term strategies.

## 1. Introduction

Benign prostatic hyperplasia (BPH) is a histological diagnosis that affects the majority of men over 50. The primary drivers of BPH and benign prostatic enlargement (BPE) are age and the presence of androgens. The androgenic effects on the prostate are primarily mediated by dihydrotestosterone (DHT), which is converted from testosterone by the enzyme 5α-reductase [1]. Two isoforms of this enzyme exist: type 1, mainly expressed in the skin and liver, and type 2, predominantly expressed in the prostate.

5-ARIs promote apoptosis of prostate epithelial cells [2], leading to a prostate volume reduction of 18–28% and a ~50% decrease in serum PSA after 6–12 months of treatment. These effects may be more pronounced with long-term use [3]. Two 5α-reductase inhibitors (5-ARIs) are approved: finasteride, which selectively inhibits type 2; and dutasteride, which inhibits both isoforms [4].

Dutasteride achieved greater DHT suppression over 6 months compared to finasteride, reducing serum DHT by ~94%, versus 70% with finasteride [4]. Finasteride lowers intraprostatic DHT by 85–90%, while dutasteride achieves a 97–99% reduction [5].

The dilemma between dutasteride and finasteride as a treatment option was addressed in a recent review. Although the overall quality of data and the strength of evidence remain limited, dutasteride may perform better in terms of reducing the likelihood of prostate surgery and AUR [4].

## 2. Methodology

A comprehensive narrative review of the literature was conducted to evaluate the current role of 5α-reductase inhibitors (5-ARIs), both as monotherapy and in combination regimens, in the management of benign prostatic hyperplasia (BPH) and lower urinary tract symptoms (LUTS). A structured search of the PubMed/MEDLINE database and Google Scholar was performed to identify relevant studies published in English up to March 2026. Search terms included combinations of “benign prostatic hyperplasia”, “5-alpha reductase inhibitors”, “5-ARI”, “finasteride”, “dutasteride”, “alpha-blockers”, “combination therapy”, “acute urinary retention”, “BPH progression”, “prostate surgery”, “sexual dysfunction”, and “prostate cancer prevention”. Additional relevant articles were identified through manual screening of reference lists from key publications and review articles.

Priority was given to randomized controlled trials, systematic reviews, meta-analyses, large observational studies, and guideline-relevant publications assessing the efficacy, safety, and long-term outcomes of 5-ARI therapy. Particular emphasis was placed on landmark studies including MTOPS, PLESS, CombAT, SMART-1, and CONDUCT. The selected literature, presented in detail in Table 1, was narratively synthesized to provide a clinically oriented overview of the effects of 5-ARIs on symptom control, prostate volume reduction, prevention of acute urinary retention and BPH-related surgery, optimization of combination therapy strategies, and treatment-related adverse events. Due to the narrative nature of the review, no formal quality assessment or quantitative meta-analysis was performed.

## 3. 5-ARIs Monotherapy

5-ARIs as monotherapy are effective in reducing prostate volume and improving lower urinary tract symptoms (LUTS) in men with BPH, particularly in those with larger prostates [6].

Trials have consistently demonstrated the effectiveness of 5-ARI monotherapy in reducing prostate size, improving LUTS, and lowering the risk of AUR and the need for surgery. In the MTOPS study, finasteride monotherapy was shown to reduce the risk of long-term clinical progression compared to placebo (17% vs. 10%, *p* < 0.001) [7]. Similarly, the CombAT study confirmed that dutasteride alone provides sustained improvements in symptom scores and reduces the risk of AUR and surgery over a four-year period [8]. These outcomes highlight the disease-modifying potential of 5-ARIs, making them the first-line option for patients with progressive BPH, enlarged prostates and at higher risk of progression.

Although the onset of symptom relief with 5-ARIs is significantly slower than that observed with alpha-blockers, their long-term benefits—such as reduction in prostate volume and prevention of complications—justify their use in selected patients who would like to avoid the side-effects of α-blockers. As monotherapy, 5-ARIs offer a well-tolerated treatment option in men with larger prostates at risk of disease progression [9].

## 4. The Ideal Patient for Combination Therapy

Combination therapy for male LUTS aims at tackling both voiding and storage symptoms when combining an α-blocker with an antimuscarinic, whereas the combination of an α-blocker with a 5-ARI aims to provide prompt symptom relief while reducing prostate size and risk of deterioration of LUTS [6].

The MTOPS [7] and CombAT [8] studies and the earlier PLESS study [10] have pictured the ideal candidate for combination therapy with an α-blocker and a 5ARI as a man with moderate to severe LUTS due to BPH, who is at risk for disease progression. Disease progression was defined as worsening of LUTS, urinary retention or need for surgery, with an increase in symptom score of at least 4 points being the most common event of progression [7].

Risk factors for disease progression include increased prostate volume and elevated serum PSA levels, both of which reflect a greater androgen-dependent prostatic burden and a higher likelihood of future clinical deterioration. Clinical trials and guideline-based recommendations commonly use prostate volume thresholds of ≥30–40 mL and PSA levels ≥1.5 ng/mL to identify men at increased risk for acute urinary retention (AUR), symptom progression, and BPH-related surgery [7,8,10]. In particular, PSA has been shown to correlate with prostate volume and may serve as a practical surrogate marker of prostatic enlargement in routine clinical practice. Men meeting these criteria derive the greatest benefit from combination therapy with an α-blocker and a 5-ARI, especially regarding long-term prevention of disease progression. An important consideration is treatment duration of at least 12 months, as the preventive benefits of combination therapy on AUR and BPH-related surgery become more evident with sustained treatment. In this setting, dual therapy provides superior long-term outcomes compared with monotherapy [7,8].

## 5. Combination Therapy from the Start Versus Add-On Therapy

The decision of when to initiate 5-ARI therapy in men with BPH already receiving α-blockers can significantly influence treatment outcomes. Emerging evidence suggests that starting 5-ARI therapy earlier may help reduce the risk of clinical progression, AUR, and prostate surgery [19,20]. Data from a large health database were used to assess the clinical and economic impacts of early versus delayed initiation of 5-ARI therapy in men already on α-blockers. The results indicated that early addition of 5-ARIs (within 30 days) led to lower rates of clinical progression (12.8% vs. 17.4%), AUR (10.2% vs. 13.8%), and prostate-related surgery (5% vs. 7%) compared to delayed addition (31 to 180 days). Additionally, early combination therapy was associated with reduced BPH-related medical costs [19].

Naslund et al. compared clinical outcomes between patients who initiated 5-ARI therapy within 30 days of starting α-blocker therapy (early cohort) and those who began 5-ARI therapy more than 30 days after starting α-blocker therapy (late cohort). The study found that each 30-day delay in initiating 5-ARI therapy was associated with an increased likelihood of overall clinical progression (average 21.1%), AUR (average 18.6%), and prostate-related surgery (average 26.7%) [20].

More evidence in favor of early start of combination therapy was provided by a later study by D’Agate et al. The authors, utilizing a time-to-event model based on pooled data from 10,238 patients included in the dutasteride trials, demonstrated significant differences (*p* < 0.001) when a switch to combination treatment occurred ≥6 months from initiation of treatment with an α-blocker. The study showed that at 4 years follow-up, the risks of AUR and BPH surgery were 4.6% vs. 11.3% for patients receiving immediate combination treatment versus those who switched to combination after 24 months [39].

However, these findings should be interpreted with caution, as much of the evidence supporting early initiation of combination therapy derives from retrospective observational studies, administrative claims databases, or post hoc modeling analyses rather than prospective randomized trials. Such study designs are inherently susceptible to selection bias, confounding factors, and differences in baseline patient characteristics, which may influence treatment outcomes independently of treatment timing. Furthermore, variations in definitions of “early” versus “delayed” therapy and differences in follow-up duration across studies may limit direct comparability. Therefore, although the available data suggest a potential benefit of earlier introduction of 5-ARIs in selected high-risk patients, further prospective randomized studies are needed to better define the optimal timing of combination therapy initiation.

## 6. Withdrawal of α-Blocker

The rationale for α-blocker discontinuation following combination therapy with 5-ARIs is to maintain symptom control while reducing medication burden and minimizing adverse effects. As 5-ARIs progressively exert their therapeutic effect on prostate volume and disease progression, continued use of α-blockers may become unnecessary in selected patients, particularly those with mild-to-moderate baseline symptoms, satisfactory symptom improvement after combination therapy, and no history of significant progression-related complications [11,12,21,22,23]. In contrast, patients with severe baseline LUTS, large residual symptom burden, or persistent voiding symptoms may be less suitable candidates for α-blocker withdrawal.

The effects of discontinuing α-blockers following 6–9 months of combination therapy with 5-ARIs have been evaluated in both a randomized controlled trial and an open-label multicenter study [21,22]. In the first study, patients receiving tamsulosin plus dutasteride were assessed after tamsulosin withdrawal at six months; 77% experienced no symptom deterioration. However, among patients with severe baseline symptoms (IPSS > 20), only 56% experienced no symptom deterioration, indicating that continued α-blocker therapy may still be necessary in this subgroup [21].

The second trial assessed finasteride monotherapy outcomes at 3 and 9 months following withdrawal of an α-blocker after 9 months of combination therapy. Improvements in LUTS persisted at both time points, with IPSS changes of 1.24 and 0.4, respectively. Nonetheless, study limitations include relatively short treatment and duration of follow-up post-discontinuation [22].

The SMART-1 trial investigated the effects of discontinuing tamsulosin after 24 weeks of combination therapy with dutasteride. Results showed that although there was a modest increase in symptom scores after α-blocker withdrawal, the majority of patients maintained acceptable symptom control with dutasteride monotherapy alone [11]. Similarly, the CONDUCT study compared early combination therapy with deferred α-blocker use in patients receiving dutasteride and found that many men did not require long-term α-blocker use, particularly those with less severe baseline symptoms [12]. These findings were further supported by a systematic review, which concluded that discontinuing α-blocker therapy after combination treatment with a 5-ARI had no significant effect on LUTS in either the short or long term [23].

Overall, current evidence suggests that α-blocker withdrawal may be considered in carefully selected patients who achieve stable symptom control after combination therapy, particularly those with moderate baseline LUTS and favorable clinical response. However, patients with severe baseline symptoms or persistent voiding complaints may derive greater benefit from continued combination treatment.

## 7. Impact of 5ARIs on Progression

α1-blockers are known to improve LUTS without, however, any interference on the natural history of BPH. A retrospective analysis of 316 patients evaluated the long-term risk of re-treatment in patients on alpha-blockers and the parameters that influence this risk [13].

The percentage of patients who received any form of re-treatment during follow-up was 38% after 3 years and 54% after 5 years. Patients with prostates larger than 40 mL were more likely to receive re-treatment within 5 years than those with smaller prostates (48% vs. 72%).

5-ARIs significantly reduce the long-term (>1 year) risk of AUR and the need for surgical intervention [7,11,14]. The results from the *Proscar™* Long-Term Efficacy and Safety Study (PLESS) on the risk of AUR according to baseline prostate volume confirmed the findings of the Olmsted community study that showed a three-fold higher risk of AUR in men with larger (>30 mL) versus smaller (≤30 mL) prostates [15].

In the PLESS trial, finasteride reduced the relative risk of AUR by 57% and of surgery by 55% over four years, with absolute risk reductions of 4% and 7%, respectively [14]. Similarly, the MTOPS study showed a 68% relative reduction in AUR risk and a 64% reduction in need for surgery, with absolute reductions of 2% and 3% [7].

A pooled analysis of three RCTs with two-year follow-up reported a 57% relative risk reduction for AUR and 34% for surgery in patients with moderate LUTS, with absolute reductions of 2% for both outcomes [16]. Dutasteride has also shown similar benefits in lowering AUR and surgery risk, supported by trials demonstrating favorable changes in urodynamic parameters [17,18].

Both the MTOPS and CombAT trials demonstrated the superiority of combination therapy over monotherapy in preventing clinical progression, defined by an increase in IPSS (≥4 points), AUR, incontinence, or a >50% rise in serum creatinine. In MTOPS, combination therapy with finasteride and doxazosin reduced the risk of long-term progression, primarily driven by symptom worsening, by 66% versus placebo, outperforming monotherapy with either finasteride (34%) or doxazosin (39%) [7]. Only regimens including finasteride (alone or in combination) significantly decreased the incidence of AUR and BPO-related surgery.

Similarly, the CombAT study showed that combination therapy with dutasteride and tamsulosin significantly lowered the relative risk of AUR by 68%, BPO-related surgery by 71%, and symptom deterioration by 41% compared to tamsulosin alone over four years [8]. Thirteen patients would need to be treated for four years to prevent one case of AUR and/or surgery, with an absolute risk reduction of 7.7%.

The CONDUCT trial, an RCT involving 742 men, compared a fixed-dose combination of dutasteride and tamsulosin with a watchful waiting (WW) strategy plus lifestyle advice and potential tamsulosin initiation. The combination therapy yielded a more substantial and sustained IPSS reduction at 24 months (5.4 vs. 3.6 points). It also reduced the relative risk of clinical progression by 43.1% versus WW, with an absolute risk reduction of 11.3% (NNT = 9) [11].

A recent meta-analysis by Cormac M. Hehir et al. suggested that preoperative 5-ARI therapy may reduce perioperative bleeding during TURP, especially in patients with enlarged prostates and increased bleeding risk [40]. However, the perioperative use of 5-ARIs in surgical patients was beyond the scope of the present review and was therefore not analyzed in detail.

## 8. Safety and Adverse Events

5-ARIs’ adverse effects are primarily related to sexual function. The most commonly reported adverse events include reduced libido and erectile dysfunction (ED), while ejaculation disorders, such as retrograde ejaculation, anejaculation, and decreased semen volume, occur less frequently [3,7,8,24]. Their impact on quality of life can be substantial, particularly in younger men or those more concerned about sexual side effects. Furthermore, a meta-analysis evaluating the effects of medical treatments for LUTS and BPH on ejaculatory function revealed that combination therapy with α-blockers and 5-ARIs increased the risk of ejaculatory dysfunction three-fold compared to either monotherapy [25].

In addition to sexual dysfunction, recent evidence has raised concerns regarding the psychological consequences of treatment with 5-ARIs. A large Swedish cohort study demonstrated an increased risk of depression in men treated with either finasteride (hazard ratio [HR] 1.61) or dutasteride (HR 1.68). However, the same study found no long-term association between the use of 5ARIs and increased risk of dementia or suicide [26]. These findings highlight the importance of careful patient selection when initiating 5-ARI therapy, particularly in individuals with pre-existing psychiatric vulnerabilities or significant concerns about sexual side effects.

### 8.1. Does Sexual Function Always Recover After Discontinuation of 5ARIs?

Sexual adverse effects including decreased libido, erectile dysfunction (ED), and reduced ejaculatory volume are well-documented adverse events. Clinical trials have shown that the majority of these effects are reversible upon discontinuation of therapy. In the PCPT and REDUCE trials, sexual dysfunction was reported more frequently in the treatment groups, but most cases resolved either spontaneously or after cessation of the drug, suggesting a primarily dose- and duration-dependent relationship [27,28].

Nevertheless, reports of persistent sexual symptoms after discontinuation of 5-ARIs have generated ongoing debate. A condition referred to as post-finasteride syndrome (PFS) has been described mainly in observational studies, retrospective analyses, and case series involving younger men treated with finasteride for androgenic alopecia rather than BPH [29,30]. Reported symptoms include persistent ED, reduced libido, genital sensory changes, and psychological distress. However, the available evidence remains limited by small sample sizes, retrospective design, selection bias, lack of appropriate controls, and difficulty establishing causality. While some studies suggest that a subset of susceptible individuals may experience prolonged symptoms [31], the true prevalence, pathophysiology, and direct causal relationship with 5-ARI exposure remain incompletely understood and controversial.

Importantly, psychological factors, including anxiety and depression, may contribute to or exacerbate perceived sexual dysfunction. Some researchers have proposed a nocebo effect in susceptible individuals, although this does not fully explain the persistence of symptoms in all cases [31]. Despite ongoing debate, regulatory agencies such as the U.S. Food and Drug Administration (FDA) and the European Medicines Agency (EMA) have acknowledged reports of persistent sexual adverse effects and updated product labeling to reflect this possibility [41].

Adverse effects associated with both drug classes have been observed in patients undergoing combination therapy [7,8,11]. These adverse events are generally consistent with the known side effects of α1-blockers and 5-ARIs. The occurrence of these events was notably more frequent in individuals receiving combination therapy. The MTOPS trial indicated that the incidence of treatment-related adverse events was higher in the first year of combined doxazosin and finasteride therapy [32].

**5-ARIs in prostate cancer prevention**

Two landmark randomized controlled trials, PCPT and REDUCE, demonstrated that finasteride and dutasteride reduced the overall incidence of prostate cancer, primarily by decreasing the detection of low-grade tumors (GS ≤ 6) [27,28]. However, both studies also reported a higher detection rate of high-grade tumors, raising concerns regarding the role of 5-ARIs in chemoprevention. Subsequent analyses suggested that this finding may partly reflect detection bias related to prostate volume reduction and improved biopsy sensitivity [33].

Importantly, both trials were conducted before the widespread use of mpMRI-guided biopsy, limiting the applicability of their findings to contemporary practice. Therefore, a clear recommendation supporting routine use of 5-ARIs for prostate cancer prevention cannot currently be made [34].

A more recent study conducted by Morote et al. evaluated the impact of 5-alpha reductase inhibitors (5-ARIs) on mpMRI findings and prostate cancer detection. The study demonstrated that 5-ARI exposure did not significantly impair PI-RADS-based detection of clinically significant prostate cancer [42].

**5-ARIs for men on active surveillance**

Recent meta-analytic evidence suggests that 5-ARIs may reduce the risk of disease progression in men undergoing active surveillance for prostate cancer, with a favorable safety profile compared to other nonsurgical interventions [35]. However, further prospective studies are needed before routine use in this setting can be universally recommended.

### 8.2. Additional Risks Associated with 5-ARIs

Gynecomastia, characterized by breast or nipple tenderness, occurs in 1–2% of individuals undergoing treatment. The cardiovascular safety profile of 5-ARIs, especially dutasteride, remains a subject of ongoing discussion [36]. Population-based investigations conducted in Taiwan and Ontario found no significant correlation between 5-ARI usage and an increased risk of cardiovascular events [36,37]. A British-Taiwanese cohort study highlighted an elevated risk of type II diabetes in men using 5-ARIs compared to those treated with tamsulosin, although the risk did not differ significantly between dutasteride and finasteride [38].

**Practical considerations:** The use of 5-ARIs is indicated for men with moderate-to-severe LUTS and an enlarged prostate (greater than 40 mL) and/or elevated PSA levels (above 1.4–1.6 ng/mL). These agents help mitigate the risk of AUR and reduce the need for surgical intervention. Due to their gradual onset of action, 5-ARIs are not suitable when immediate symptom relief is sought. It is important to factor in their effect on PSA levels when considering PCa screening.

A concise comparison between finasteride and dutasteride regarding enzyme selectivity, degree of DHT suppression, pharmacokinetic properties and adverse effect profile is presented in Table 2, highlighting the main clinical differences that may influence individualized treatment selection in men wih BPH. Data adapted from references [4,5,7,8,14,24,25].

**Practical considerations for patient counseling**

Appropriate patient counseling before initiation of 5-ARI therapy is essential to improve adherence, set realistic expectations, and facilitate shared decision-making. Patients should be informed that symptom improvement is gradual and may require several months of treatment, while potential adverse effects most commonly involve sexual function, including reduced libido, erectile dysfunction, and ejaculatory changes. Importantly, available evidence suggests that the majority of sexual adverse events are mild and reversible after discontinuation of therapy.

Clinicians should also discuss the possibility of psychological influences on treatment perception and adverse event reporting. A nocebo effect, whereby negative expectations contribute to symptom development or amplification, has been proposed in association with 5-ARI therapy. Therefore, counseling should remain balanced and evidence-based, adequately informing patients about potential risks without unnecessarily increasing anxiety or treatment avoidance. Individualized counseling is particularly important in younger men and in patients with pre-existing sexual or psychological concerns.

## 9. Conclusions

When compared to α1-blockers or 5-ARI monotherapy, combination therapy provides superior improvements in LUTS and uroflowmetry parameters and is more effective in preventing disease progression. However, this treatment regimen is associated with a higher incidence of adverse events. Therefore, combination therapy should primarily be prescribed for men with moderate-to-severe LUTS who are at risk of disease progression—such as those with increased prostate volume, elevated PSA levels, advanced age, high post-void residual (PVR) volume, and reduced Qmax. It should be reserved for individuals expected to adhere to long-term treatment (greater than twelve months). In cases of moderate LUTS, discontinuation of the α1-blocker after six months may be considered. Given the delayed onset of action and the potential for sexual adverse effects, effective patient counseling is crucial to ensure compliance and appropriate expectations.

## Figures and Tables

**Table 1:** Summary of studies included. 5ARIs: 5-alpha reductase inhibitors BPH: Benign Prostatic Hyperplasia DHT: dihydrotestosterone AUR: acute urinary retention ED: erectile dysfunction EjD: ejaculatory dysfunction PCa: prostate cancer AS: active surveillance CVD: cardiovascular disease CV: cardiovascular; T2DM: type 2 diabetes mellitus.

Author/Year	Type of Study	Patients (n)	Outcome Searched	Result	Comment
			**Scientific rationale**		
Andriole G, 2004 [1]	Review	n/a (Review)	Scientific rationale for 5ARIs in BPH	DHT plays key role; 5ARIs mechanistically justified	Foundational rationale for therapy
Clark RV, 2004 [5]	Clinical trial	399 men	DHT suppression by dutasteride	Marked DHT suppression demonstrated	*p* < 0.001, safety profile similar to placebo
Rittmaster RS, 1996 [2]	Clinical trial	21 men	Histological effects of finasteride	Evidence of atrophy and apoptosis in prostate tissue	Mechanistic evidence of action
**Clinical effects and effects on BPH progression**					
Naslund MJ, 2007 [3]	Review	n/a (Review)	Efficacy and safety of 5ARIs	5ARIs reduce symptoms, prostate volume, and risk of AUR/surgery	Summarizes available data up to 2007
Pirozzi L, 2015 [4]	Review	n/a (Review)	Comparison of dutasteride vs. finasteride, risk of surgery/AUR	Both effective, dutasteride may provide greater suppression	No large head-to-head outcome RCTs
Sountoulides P, 2015 [6]	Review	n/a (Review)	Combination pharmacological treatments	Combination improves LUTS outcomes	Short editorial-like review
McConnell JD, 2003 [7]	RCT (MTOPS)	3047 men	Progression of BPH with doxazosin, finasteride, or both	Combination most effective; finasteride reduced risk of AUR and surgery	Landmark trial in BPH
Roehrborn CG, 2010 [8]	RCT (CombAT)	4844 men	Dutasteride + tamsulosin vs. monotherapy, time to first AUR or BPH-related surgery. BPH clinical progression, symptoms, Q(max), prostate volume, safety, and tolerability.	Combination superior to either alone for symptoms and progression	Supports combination therapy in large prostates, in men with moderate to severe LUTS
Kim JH, 2018 [9]	Meta-analysis	23 RCTs (~27,000 men)	5ARI monotherapy efficacy and safety	Significant improvement in LUTS, reduced prostate volume, acceptable safety	Up-to-date pooled evidence, need for more head-to-head comparisons
Roehrborn CG, 1999 [10]	RCT (PLESS)	3040 men	Finasteride vs. placebo, 4 years	Reduced risk for AUR and surgery, PSA > 1.4 ng/mL and volume predicted outcomes	Key long-term evidence
Roehrborn CG, 2008 [11]	RCT (CombAT 2y)	4844 men	Combination vs. monotherapy	Combination superior at 2 years	Interim results before 4y data
Roehrborn CG, 2015 [12]	RCT (CONDUCT)	742 men	Fixed-dose dutasteride+tamsulosin vs. watchful waiting	Combination reduced risk of progression, better LUTS	Early intervention beneficial
De La Rosette JJ, 2002 [13]	Observational	2200 men	Re-treatment risk in α-blockers monotherapy. Tamsulosin has a markedly lower re-treatment percentage than alfuzosin and terazosin.	High long-term retreatment rates	α-blockers do not modify disease course
McConnell JD, 1998 [14]	RCT	3040 men	Finasteride vs. placebo. Qmax, BPH progression	Increased Qmax, reduced AUR and surgery risk	Foundational efficacy trial
Jacobsen SJ, 1997 [15]	Cohort study	2097 men	Risk factors for AUR	Larger prostate/PSA risk	Natural history data
Andersen JT, 1997 [16]	RCT	895 men	Finasteride’s impact on AUR and surgery	Reduced both outcomes significantly	Supports efficacy
**Urodynamic effects**					
Kirby RS, 1993 [17]	Pilot study	15 men	Urodynamic effects of finasteride	Improved pressure/flow parameters	Small study
Tammela TL, 1995 [18]	Clinical trial	46 men	Urodynamics and symptoms, long-term finasteride	Improved urodynamic measures, symptom relief	Longer-term physiologic evidence
**Delayed 5-ARI therapy**					
Kruep EJ, 2011 [19]	Observational (claims data)	Claims data (~17,000 men)	Early vs. delayed 5ARI therapy	Early initiation reduced progression, costs	Retrospective, economic focus
Naslund M, 2009 [20]	Observational	Claims data (~7000 men)	Impact of delayed 5ARI	Delays risk of AUR and surgery within 12 months	Supports timely initiation
**a-blocker discontinuation**					
Barkin J, 2003 [21]	RCT	327 men	α-blocker withdrawal after dutasteride	Most men maintained control on dutasteride monotherapy	Supports step-down strategy
Nickel JC, 2008 [22]	RCT	395 men	Finasteride monotherapy 9 months after α-blocker cessation	Control of LUTS maintained	Supports discontinuation approach
van der Worp H, 2019 [23]	Systematic review/meta-analysis	19 studies (~3500 men)	Discontinuation of α-blockers after combination therapy	Discontinuation feasible in most after combination	Useful practical implication
**Side-effects**					
Corona G, 2017 [24]	Systematic review/meta-analysis	17 studies (~17,000 men)	Sexual dysfunction with 5ARIs	risk of ED, libido, ejaculatory issues	Large synthesis, highlights adverse effects
Gacci M, 2014 [25]	Systematic review/meta-analysis	24 studies (~22,000 men)	Impact of LUTS therapies on ejaculation	Combination therapies 3× risk of EjD compared to monotherapies	Comparative to other treatments
Garcia-Argibay M, 2022 [26]	Cohort study	>200,000 men (2 cohorts)	5ARIs and risk of dementia/depression	Association with risk	Observational, but needs caution
Thompson IM, 2003 [27]	RCT (PCPT)	18,882 men	Finasteride for PCa prevention	overall PCa incidence, high-grade tumors	Controversial findings
Andriole GL, 2010 [28]	RCT (REDUCE)	8121 men	Dutasteride for PCa risk	low-grade cancer risk, possible high-grade	FDA safety concerns
Irwig MS, 2012 [29]	Case series	71 men (case series)	Persistent sexual side effects post-finasteride	Reported long-term dysfunction	Limited evidence, controversial
Traish AM, 2020 [30]	Review	n/a (Review)	Post-finasteride syndrome	Describes persistent adverse effects	Critical view, debated
Belknap SM, 2015 [31]	Meta-analysis	34 trials (~11,000 men)	Adverse event reporting in alopecia finasteride trials	Under-reporting suspected	Highlights bias issues
Kaplan SA, 2016 [32]	RCT (MTOPS adverse events)	3047 men	Time course of side effects	Most occur early in treatment	Useful for patient counseling
Thompson IM, 2006 [33]	Secondary analysis	18,882 men	Effect on PSA sensitivity	Finasteride improves PSA specificity	Diagnostic implication
Liss MA, 2018 [34]	Review	n/a (Review)	PCa prevention with 5ARIs	Controversies, modest prevention benefit	Balanced appraisal
Matsukawa A, 2024 [35]	Systematic review/meta-analysis	22 studies (~11,000 men)	Non-surgical interventions in AS PCa	5ARIs may delay progression	Recent evidence, ongoing debate
Hsieh TF, 2015 [36]	Cohort study	19,735 men	CVD risk with 5ARIs	No increased risk	Supports safety
Skeldon SC, 2017 [37]	Cohort study	72,000 men	CV safety of dutasteride	No excess CV risk	Population-based reassurance
Wei L, 2019 [38]	Cohort study	~30,000 men (UK database)	5ARIs and type 2 diabetes risk	risk of T2DM	New metabolic concern

**Table 2:** Comparison of Finasteride vs. Dutasteride.

Parameter	Finasteride	Dutasteride
Enzyme inhibition	Selective inhibition of type 2 5α-reductase	Dual inhibition of type 1 and type 2 5α-reductase
Standard dose for BPH	5 mg once daily	0.5 mg once daily
Serum DHT suppression	~70% reduction	~90–95% reduction
Intraprostatic DHT suppression	~85–90% reduction	~95–99% reduction
PSA reduction	~50% after 6–12 months	~50% after 6–12 months
Onset of clinical effect	Gradual (months)	Gradual (months)
Half-life	~6–8 h	~5 weeks
Time to elimination after discontinuation	Days	Several months
Evidence for reducing AUR/surgery	Strong evidence (PLESS, MTOPS)	Strong evidence (CombAT and other trials)
Sexual adverse effects	Reduced libido (3–6%), ED (5–8%), ejaculatory dysfunction (2–4%)	Reduced libido (4–7%), ED (6–9%), ejaculatory dysfunction (3–5%)
Main clinical advantage	Lower cost, shorter half-life	More potent and sustained DHT suppression
Main clinical limitation	Less complete DHT suppression	Long half-life may prolong adverse effects after discontinuation

## Data Availability

No new data were created or analyzed in this study.
